# As human societies urbanize, so does ecology; taxonomic, geographic, and other research trends in urban vertebrate ecology

**DOI:** 10.1002/ece3.11439

**Published:** 2024-05-20

**Authors:** Travis A. Rainey, Alaini C. Schneider, Carson J. Pakula, Bradley J. Swanson

**Affiliations:** ^1^ Department of Biology Central Michigan University Mount Pleasant Michigan USA; ^2^ Western EcoSystems Technology, Inc. Golden Valley Minnesota USA; ^3^ Savannah River Ecology Laboratory University of Georgia Aiken South Carolina USA; ^4^ Warnell School of Forestry and Natural Resources University of Georgia Athens Georgia USA

**Keywords:** biodiversity, geographic, non‐native, piecewise regression, temporal trends, terrestrial vertebrate, threatened, wildlife

## Abstract

The threat to biodiversity posed by urban expansion is well researched and supported. Since the late 1990s, the field of urban ecology has been expanding along with the developed landscapes it studies. Past reviews have shown unequal publication rates in urban ecology literature for taxonomic groups and research locations. Herein, we explore differences in the publication rate of urban studies by vertebrate groups, but also expand on previous investigations by broadening the scope of the literature searched, exploring trends in subtopics within the urban wildlife literature, identifying geographic patterns of such publications, and comparing the rate at which non‐native and threatened and endangered species are studied in urban settings. We used linear and segmented regression to assess publication rates and Fisher's exact tests for comparisons between groups. All vertebrate groups show an increasing proportion of urban studies through time, with urban avian studies accelerating most sharply and herpetofauna appearing to be understudied. Non‐native mammals are more studied than non‐native birds, and threatened and endangered herpetofauna and mammals are more likely to be studied than threatened and endangered birds in urban areas. The plurality of urban wildlife studies are found in North America, while there is a dearth of studies from Africa, Asia, and South America. Our results can help inform decisions of urban ecologists on how to better fill in knowledge gaps and bring a greater degree of equity into the field.

## Graphical abstract

Since the late 1990s, the field of urban ecology has been expanding along with the developed landscapes it studies. Herein, we explore differences in the publication rate of urban studies by vertebrate groups, but also expand on previous investigations by broadening the scope of the literature searched, exploring trends in subtopics within the urban wildlife literature, identifying geographic patterns of such publications, and comparing the rate at which non‐native and threatened and endangered species are studied in urban settings.

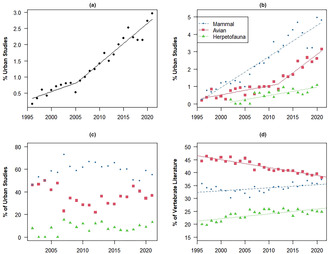

## 1 INTRODUCTION

Although worldwide urbanization is predicted to continue reducing biodiversity in regions where expansive land conversions are expected in the coming decades (Fenoglio et al., 2020; Li et al., 2022; McKinney, 2002; Miller & Hobbs, 2002), the number of ecological studies investigating urban areas is lower than expected, based on the proportion of urbanized land (Martin et al., 2012). Globally, urban areas only account for about 3% of landcover (Liu et al., 2014). However, as of 2021, 56% of the human population was estimated to live in urban settings (United Nations, 2022), and projections indicate 68% of the global population is expected to live in urban areas by 2050 (United Nations Department of Economic and Social Affairs, 2018). Urban development and its associated fragmentation can limit gene flow (Delaney et al., 2010) and species richness (Theodorou et al., 2020), and can provide non‐native species with opportunities to colonize disturbed landscapes and outcompete native species (Cadotte et al., 2017; Dearborn & Kark, 2010). These problems are exacerbated as cities are typically located in biodiverse places (Kowarik, 2011). While natural ecosystems are notably altered in urban areas, studying urban ecosystems can help minimize the impact of current and projected urbanization on biodiversity. The demand for such studies is especially high in developing countries, where the greatest number of people are projected to move to urban centers (United Nations, 2022).

As human society urbanizes, urban ecology research can help policymakers implement more effective and sustainable development strategies, which benefit from prioritizing biodiversity (Blicharska et al., 2019). Thus, while humankind continues to develop more of the natural world, we are also learning how to do so more responsibly. In addition to sustainable growth, the study and management of these urban ecosystems can help protect populations of rare species (Kowarik, 2011; Miller & Hobbs, 2002), increase connectivity between natural populations (Beninde et al., 2015; Miller & Hobbs, 2002), educate and connect people with nature and the importance of conservation, promote ecological restoration in disturbed areas (Miller & Hobbs, 2002), harness ecosystem services, and improve human well‐being and public health (Dearborn & Kark, 2010; Kowarik, 2011).

Despite the growth in urban ecology studies (Collins et al., 2021; Magle et al., 2012; Rega‐Brodsky et al., 2022) knowledge gaps exist (Knapp et al., 2021; Rega‐Brodsky et al., 2022) and ecologists continue to study more natural areas relative to developed areas (Martin et al., 2012; Ruggieri‐Mitchell, 2023). Ruggieri‐Mitchell (2023) argued that a greater proportion of conservation and ecological research should be focused on urban study areas, where wildlife populations are expected to face greater conservation challenges. Additionally, as urban ecology grows, it is integral to identify knowledge gaps and biases within the literature to identify conservation concerns and better understand anthropogenic influence in developing areas (see Collins et al., 2021; Magle et al., 2012).

Focusing on terrestrial vertebrates (hereafter, vertebrates), we analyzed trends in the publication rate of urban studies each year since the late 1990s, when urban ecology began to have a measured presence in the literature (Magle et al., 2012; Rega‐Brodsky et al., 2022). Terrestrial vertebrates are the most studied wildlife taxa in urban areas (Magle et al., 2012), and thus provides the most robust dataset for assessing publication trends in urban studies. As the number of ecology and conservation publications has steadily risen across all subdisciplines over the past 100 years (Anderson et al., 2021; Kim et al., 2018), we assessed the proportion of urban studies within the broader vertebrate conservation and ecology literature. Previous studies have documented a proportional rise in urban ecology studies, but Magle et al. (2012) only investigated 16 handpicked journals, and although Collins et al. (2021) did not restrict their search to specific journals, they only considered publications between 2011 and 2020. Additionally, Rega‐Brodsky et al. (2022) provided a detailed investigation of trends in urban ecology between 1990 through mid‐2018 but specifically focused on publications studying biodiversity and biotic communities in urban areas, which may have missed certain urban ecology topics. We expanded these previous inquiries by attempting to capture a greater totality of the urban vertebrate ecology literature by not restricting our search to specific journals and including publications from a longer time period (relative to Magle et al., 2012 and Collins et al., 2021) while also including single‐species and various study types not under the biodiversity umbrella (relative to Rega‐Brodsky et al., 2022; e.g., methods or wildlife disease papers) to fully encompass trends in urban vertebrate literature.

Along with assessing the proportion of urban studies within the overall vertebrate literature, we assessed differences in publication rates of urban studies between vertebrate groups, and we used breakpoint analysis (e.g., piecewise or broken‐stick regression) to determine whether these publication rates changed over time. In this technique, segmented linear models are fit to the data, and the model with the most appropriate number of segments is determined. This process allows changes in rates to be easily identified as each significant rate change will produce another breakpoint and line segment.

We also explored trends in subtopics within the urban wildlife literature and how vertebrate groups compare regarding subtopics, compared differences among taxa with respect to representation in urban studies based on the International Union for Conservation of Nature (IUCN) listing status (hereafter, listed), non‐native species status, and characterized the geographic representation of urban vertebrate studies by continent. These assessments have previously been unaddressed entirely or not addressed at the scale of literature search we provide. We hypothesized that urban studies are growing across the breadth of vertebrate literature. We also expected there would be patterns within urban ecology that have been found within the broader ecological literature, such as underrepresentation of areas outside North America and Europe and unequal representation of taxa (Di Marco et al., 2017; Martin et al., 2012; Pyšek et al., 2008). Our findings can help identify trends and shortcomings in this growing field and suggest possible course‐correction for future focus of this growing field of research.

## 2 MATERIALS AND METHODS

### 2.1 Literature search

For our literature review, we searched the Web of Science (WOS) Core Collection for English language publications that, (A) researched a terrestrial vertebrate (limited to herpetofauna, avian, and mammalian taxa) using empirical data, and (B) included an urban study area, with the terms “urban” and “wildlife”, joined with the “AND” operator, in the title, abstract, or author keywords for the years 1995–2021. Studies with “periurban”, “suburban”, “exurban”, and other iterations of similar terms appeared in our search without the use of an asterisk, thus, our definition of “urban” includes these types of developed areas, as defined by authors. Due to occasional discrepancies between year of indexing and year of publication (for examples, see Liu, 2021), we further isolated the search results to only include papers published from 1995–2021. We refined all searches to return only peer‐reviewed academic articles to avoid inclusion of literature reviews, book reviews, gray literature, books, or other document types. Data collection consisted of scoring publications for a set of variables (see below). Scoring was generally possible by reviewing abstracts and titles, of which WOS allows large batch downloads. The full publications were evaluated when sufficient information was not able to be obtained from the abstract.

For each publication, the following variables were scored: vertebrate group(s) being studied (herpetofauna, avian, mammal), continent in which the study took place, IUCN Red List species status (we aggregated near threatened, vulnerable, endangered, critically endangered, and extinct in the wild into one “listed” group, because there were not enough studies per year on species of each status to conduct separate assessments; IUCN, 2022), whether the study organism is considered non‐native to the study area, and the research topics the study investigated. A study organism was considered non‐native if the study area did not match the continent(s) containing a species' native range, or if authors stated species were non‐native. We chose research topics based on terms which commonly appeared in the publications we evaluated, including: behavior/cognition, community composition, conservation, diet, environmental toxicology, genetics, habitat use/dispersal/movement, human–wildlife conflict, management/wildlife control, methods, morphology/physiology, occupancy, population dynamics, predator–prey relationships, public health, reproduction, and wildlife disease. If a paper studied at least one non‐native species, that paper was only scored as studying non‐native species, even if it also studied native species. Similarly, papers that studied at least one listed species were only scored as studying listed species, even if they also researched non‐listed species. Publications with multiple topics in their research objectives were included as publications in each of those areas.

To calculate yearly proportions of urban studies, we needed to estimate yearly totals of all publications, including non‐urban studies, for each vertebrate group to use as denominators. To find non‐urban papers of each vertebrate group, we searched WOS for article titles, abstracts, and author keywords with the root of the phylogenetic class name of each terrestrial group with an asterisk after the root to harness similar terms (i.e., “reptil*”, “amphib*”, “mammal*”, “avian*”; note “avian*” was used instead of “aves*”, which increased results nearly tenfold). We followed each taxonomic search term with the “NOT” logical operator and “urban*”, to return articles that did not contain forms of the word “urban” in their titles, abstracts, or author keywords. We then searched and included additional taxonomic search terms per taxonomic class for greater representation, as authors might not explicitly reference terms rooted in the class name. We chose these additional search terms by picking common nomenclature that returned at least 1000 articles for the 26‐year span. Mammal literature was comprised of the results from the following search terms: “mammal*”, “ungulat*”, “canis*”, “rodent*” and “primate*”. Avian literature was comprised of the results from the following search terms: “avian*”, “bird*”, “raptor*”, and “passerine*”. As the herpetofauna group consists of two taxonomic classes of organisms, (Amphibia and Reptilia), more additional search terms were attempted. This search consisted of the following terms: “reptil*”, “amphib*”, “snake*”, “lizard*”, “turtle*”, “frog*”, “salaman*” and “anuran*”.

We refined our WOS search for non‐urban papers to only return articles categorized as “Ecology” or “Biodiversity Conservation” studies by WOS, to exclude social science, medical, and other kind of publications which might have taxonomic terms included in their abstracts or titles but do not conform to the interests of this review. Results of these searches were then combined by vertebrate group (herpetofauna, avian, mammal), duplicate publications were removed (e.g., a publication that was returned for both “avian*” and “passerine*” searches was only included once), and yearly totals of publications for a given group were calculated. We then combined all three groups' non‐urban publications and removed duplicates to calculate yearly totals of overall vertebrate ecology and conservation studies. To calculate the proportion of urban studies within each group, as well as collectively among all groups, we then simply divided the number of urban studies by the number of urban and non‐urban studies per group per year.

### 2.2 Trends over time by taxa

We used simple linear regression models to identify nonzero slopes in the percentage of urban studies within the vertebrate wildlife literature, the percentage of studies conducted in urban study areas within each taxon's respective body of literature, the percentage of each vertebrate group's representation within the body of urban vertebrate literature, and the percentage of each vertebrate group's representation within the overall body of vertebrate literature with simple linear regression. These percentage data were analyzed per year, in a non‐cumulative fashion (i.e., each point in a regression represents the percent of studies of a given category for a given year). Simple linear models were fit using the base “stats” package in R, and all other statistics were conducted using the R programming language v. 4.1.1 (R Core Team, 2021) in the RStudio integrated development environment (RStudio Team, 2020), unless otherwise noted. The alpha level was set to *α* = .05 for all analyses, with Bonferroni corrections applied as needed. To limit stochasticity near the y‐intercept, regressions analyzing any dependent variable as the percentage of urban studies (e.g., the proportion of each vertebrate group's contribution, subtopics, study area continent within the urban literature) only considered years after 2000, as there were fewer than 10 urban studies scored per year prior to 2001.

For some of the regressions, breakpoints (i.e., inflection points) appeared evident, so a piecewise regression approach (for wildlife research examples, see Czudaj et al., 2022; McNicol et al., 2020) was implemented for testing changes in the rate of publications. To test for the existence of breakpoints (*k*) in our models and to determine the number of breakpoints, we followed the methods of Muggeo (2020), using sequential hypotheses testing with Bonferroni correction (*α* = .025) to determine *k*. For piecewise analyses, we used the R package “segmented” (Muggeo, 2008). The score test statistic (*Z*), which is more powerful than other tests for a single breakpoint (Muggeo, 2016), was first calculated to test for the existence of one breakpoint, whereby H_0_ is a simple linear model without any breakpoints. If this first test failed to reject H_0_, we determined no significant breakpoints were present. If the initial test rejected H_0_, a sequential test was performed to test for the existence of a second breakpoint, whereby H_0_ becomes *k* = 1, and H_a_: *k* > 1. This process also served to validate a statistically significant difference in the piecewise segments' slopes; breakpoint significance requires significant difference in segment slopes (Muggeo, 2008). No two‐breakpoint models were discovered during analysis, so no further testing (e.g., H_a_: *k* = 3) was necessary.

If significant breakpoints existed, piecewise models of the highest likelihood were fit to the original simple linear model by running a bootstrapping algorithm for a given range of values of the independent variable, starting from the median (Muggeo, 2008). Significant breakpoint estimates refer to the first value of the second linear segment (i.e., if a significant breakpoint was estimated to be in 2009, 2009 is the first year included in the second segment). Adjusted coefficients of determination were then calculated for piecewise models.

For all simple linear regression models (*k* = 0), as well as for all piecewise segments, *F*‐statistic (*F*) and corresponding *p* value (*p*), slope estimates (*β*), and multiple coefficients of determination (*R* ^2^) were calculated. Datapoints with corresponding residuals >2 standard deviations from the mean were treated as outliers and removed. Normality of residuals was tested with Shapiro–Wilk tests. Breakpoints for piecewise segments with non‐normally distributed residuals were rejected and simple linear regression analyses were applied.

### 2.3 Trends in urban wildlife publication topics

Yearly percentages of research topics represented in the urban wildlife literature were calculated. Via simple linear regression, we tested for trends in topic percentages over the study period. We did not include the morphology/physiology topic in regression analyses because we did not find papers on this topic until 2017. Then, we used multi‐response permutation procedures (MRPP; Berry & Mielke, 1984) with Euclidean distance metrics implemented in PC‐ORD v. 6.0 (McCune & Mefford, 2010) to test for differences in study topics among taxonomic groups, followed by pairwise comparisons. For MRPP analysis, a presence‐absence matrix was constructed, whereby columns corresponded to topics, and rows corresponded to each study.

Post‐hoc, pairwise Fisher's exact tests were conducted using the “fsmb” R package (Nakazawa, 2022), to determine if and how taxa differed for each topic, driving the overall separation of groups. Fisher's exact tests use contingency tables and are recommended for count data when samples sizes are small (Ruxton & Neuhäuser, 2010), like these topic data. A Bonferroni‐corrected significance level (*α* = .0167) was used to account for multiple comparisons.

### 2.4 Representation of listed and non‐native species, geographies

The yearly percentage of studies researching listed species, as well as the yearly percentage of studies researching non‐native species, were calculated. We took a conservative approach to non‐nativeness, scoring study species as native if the study area was on the same continent as the species' native range, unless authors specifically noted that they were studying non‐native populations. Therefore, studies of non‐native populations that were not deemed as such by authors and had study areas in the continent that contains that species native range would not have been scored as a non‐native (e.g., American Bullfrog [*Lithobates catesbeianus*] populations in the mountain west of North America). We classified domestic animals without naturally occurring populations (e.g. domestic cat [*Felis catus*]) as non‐native in our literature review regardless of study location.

We also calculated yearly percentages of urban wildlife publications with study areas in each continent, to test for trends in the proportional representation of geographies over time. We tested for non‐zero slopes for these variables over time with simple linear regression to assess whether there has been a change in the rate of publication of these variables in the urban wildlife literature. Additionally, we tested for differences in the representation of non‐native and listed species between taxonomic groups, as well as for differences in the representation of continents, using pairwise Fisher's exact test with Bonferroni correction (*α* = .0167).

## 3 RESULTS

### 3.1 Literature search

The search for urban wildlife publications between 1995 and 2021 in WOS returned 3176 documents. Of those studies returned, 1229 (38.7%) publications from 309 journals met our criteria to be scored. The first of these studies was published in 1996, so our datasets start that year. Of these 1229 urban wildlife papers, 109 (8.87%) studied herpetofauna, 433 (35.23%) studied birds, and 724 (58.91%) studied mammals (some publications researched multiple taxa, hence percentages sum to over 100%). Our search for overall conservation and ecology publications for these vertebrate groups, including studies of non‐urban areas, yielded 70,013 studies published between 1996–2021. While mammals were the most studied vertebrate group in urban settings, birds were the most studied group in our overall search, comprising 41.4% of overall vertebrate conservation and ecology literature, followed by mammals at 34.3%, and herpetofauna at 24.3%.

### 3.2 Trends over time by taxa

**FIGURE 1:**
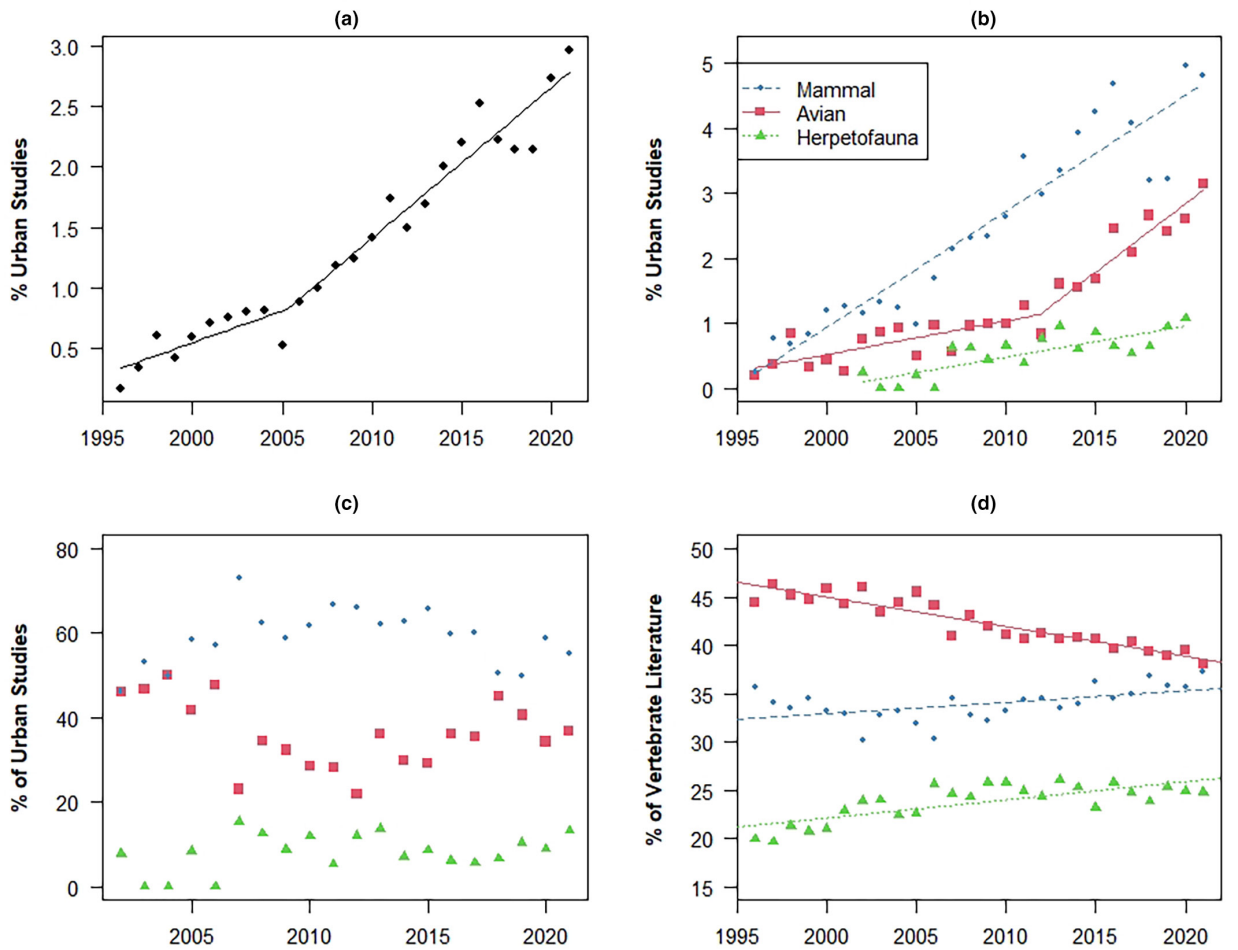
Plots for regression analyses with lines representing significant trends and significant breakpoints: (a) the trend in yearly percentage of urban studies within the broader terrestrial vertebrate literature, (b) the trend in yearly percentage of urban studies within each vertebrate group's respective literature, (c) yearly percentages of each terrestrial vertebrate group's proportional representation within the urban vertebrate literature, (d) trends for terrestrial vertebrate groups' representation within the overall terrestrial vertebrate literature (including non‐urban studies). See Table 1 for full regression output. The legend in (b) also corresponds to (c) and (d).

The percentage of urban studies within the overall vertebrate literature increased over the study period with a significant breakpoint in 2005 (*Z* = 3.037; *p* = .006, Figure 1a, Table 1), when the publication rate of urban studies increased significantly (aggregated regression of both piecewise segments: *F* = 12.393, *df* = 1, 22, *p* = .002, *R* ^2^ = 0.957; see Table 1 for segment results). A second breakpoint (Ha: k > 1) was not found (Z = −0.776; *p* = .446) in the overall urban publication rate. All taxonomic groups show an increasing proportion of urban studies (all *F* ≥ 31.292, *p* < .001; Figure 1b; see Table 1 for full regression results), although there were no significant breakpoints for either herpetofauna or mammals (both *Z* ≤ 1.167, both *p* ≥ .255). However, similar to the overall analysis, we did find a significant breakpoint in the publication rate of urban bird studies in 2012 (*Z* = 3.565, *p* < .001; aggregated regression of both piecewise segments: *F* = 33.734, *df* = 1, 22, *p* < .001, *R* ^2^ = 0.932; Figure 1b; see Table 1 for segment results), but no second breakpoint was present (*Z* = 0.007; *p* = .994).The 2012 breakpoint in the avian literature indicates an increase in urban avian research, where up to 2012, the rate of urban studies in avian literature (0.052%/year) was nearer to that of herpetofauna literature (0.047%/year) than to the mammal literature (0.178%/year), but then assumed a significantly greater rate of increase (0.210%/year), similar to that for mammals. No urban herpetofauna publications were found between 1995–2000, and 2001 and 2021 were excluded as high outliers (Figure S1). Therefore, we limited the urban herpetofauna regression to data from 2002 to 2020.

**TABLE 1:** Full regression segment results for vertebrate comparison analyses.

Regression model	*R* ^2^	*F* (*df*)	*p*_1_ (*F*)	*β* ± *SE*	W	*p*_2_ (Shapiro–Wilk)	Corresponding figure
% of vertebrate studies in urban areas (first segment; 1996–2004)	0.859	42.76 (1, 7)	<0.001*	0.076 ± 0.012	0.927	0.457	1a
% of vertebrate studies in urban areas (second segment; 2005–2021)	0.924	181.3 (1, 15)	<0.001*	0.130 ± 0.010	0.986	0.993	1a
% of herpetofauna studies in urban areas (2002–2020)	0.648	31.292 (1, 17)	<0.001*	0.047 ± 0.008	0.953	0.446	1b
% of bird studies in urban areas (first segment; 1996–2011)	0.583	19.54 (1, 14)	0.001*	0.052 ± 0.012	0.958	0.630	1b
% of bird studies in urban areas (second segment; 2012–2021)	0.866	51.63 (1, 8)	<0.001*	0.210 ± 0.029	0.948	0.641	1b
% of mammal studies in urban areas (1996–2021)	0.893	201.1 (1, 24)	<0.001*	0.178 ± 0.013	0.947	0.201	1b
% of urban studies on herpetofauna (2002–2021)	0.116	2.354 (1, 18)	0.142	0.259 ± 0.169	0.973	0.810	1c
% of urban studies on birds (2002–2021)	0.083	1.637 (1, 18)	0.217	−0.398 ± 0.311	0.955	0.456	1c
% of urban studies on mammals (2002–2021)	0.007	0.121 (1, 18)	0.7318	0.092 ± 0.265	.972	0.806	1c
% herpetofauna studies in overall literature (1996–2021)	0.567	31.38 (1, 24)	<0.001*	0.188 ± 0.034	0.954	0.289	1d
% bird studies in overall literature (1996–2021)	0.872	163.4 (1, 24)	<0.001*	−0.306 ± 0.024	0.973	0.704	1d
% mammal studies in overall literature (1996–2021)	0.277	9.176 (1, 24)	0.006*	0.121 ± 0.040	0.979	0.843	1d

*Note*: Asterisks indicate statistically significant *p* values. The two regression models with significant breakpoints (the percentage of all terrestrial vertebrate studies in urban areas, and the percentage of bird studies in urban areas) are reported by linear piecewise segments (see text for combined regression results for these regression models). The coefficient of determination (*R* ^2^), *F*‐statistic (*F*), degrees of freedom (*df*), *p* value for regression model (*p*_1_), slope (*β*) ± the standard error (*SE*) of the slope, Shapiro–Wilk test statistic for normality (*W*), and its corresponding *p* value (*p*_2_) are reported for all regression segments.

There were few publications with urban study areas published (*n* < 12) prior to 2002. Therefore, to regress each vertebrate group's contribution to the urban literature over time, we considered data from 2002–2020. The relative contribution of each taxon within the urban literature stayed constant over time (all *F* ≤ 2.354, all *p* ≥ .142; Figure 1c, see Table 1 for complete regression output). However, our assessment of these groups' representation within the overall (i.e., urban and non‐urban) vertebrate conservation and ecological literature suggests significant positive trends in the percentage of vertebrate literature researching herpetofauna (*β* = 0.188, *F* = 31.38, *df* = 1, 24, *p* < .001) and mammals (*β* = 0.121, *F* = 9.176, *df* = 1, 24, *p* = .006), while the proportion of vertebrate ecology and conservation literature studying birds significantly decreased (*β* = −0.306, *F* = 3163.4, *df* = 1, 24, *p* < .001; Figure 1d, Table 1).

### 3.3 Trends in urban wildlife publication subtopics

In assessing changes in the proportion of subtopics over the study period (Table 2), we saw four subtopics with non‐zero slopes of their relative contribution to yearly urban vertebrate literature, including: environmental toxicology (*β* = 0.397, *F* = 14.050, *df* = 1, 24, *p* < .001), diet (*β* = 0.331, *F* = 5.496, *df* = 1, 19, *p* = .030), occupancy (*β* = 0.632, *F* = 17.269, *df* = 1, 19, *p* < .001), and methods (*β* = 0.312, *F* = 10.017, *df* = 1, 19, *p* = .005). However, only environmental toxicology and occupancy publication trends had normally distributed residuals.

**TABLE 2:** Results for regression analysis of the prevalence of subtopics within urban vertebrate literature over the study period.

Topic	% of urban publications (2001–2021)	*R* ^2^	*F* (*df*)	*p*_1_ (*F*)	*β* ± *SE*	*W*	*p*_2_ (Shapiro–Wilk)
Behavior, cognition	13.88	0.111	2.376 (1, 19)	0.140	0.494 ± 0.321	0.688	<0.001
Community composition	8.0	0.119	2.577 (1, 19)	0.125	−0.303 ± 0.189	0.935	0.182
Conservation	3.8	0.125	2.713 (1, 19)	0.116	−0.259 ± 0.157	0.963	0.555
Diet	6.6	0.224	5.496 (1, 19)	0.030*	0.331 ± 0.141	0.876	0.012
Environmental toxicology	7.0	0.425	14.050 (1, 19)	<0.001*	0.397 ± 0.106	0.960	0.552
Genetics	4.3	0.128	2.789 (1, 19)	0.111	0.169 ± 0.101	0.956	0.446
Habitat use, dispersal, movement	22.3	0.002	0.029 (1, 19)	0.867	0.043 ± 0.253	0.942	0.240
Human–wildlife conflict	8.2	0.001	0.027 (1, 19)	0.871	0.034 ± 0.205	0.951	0.345
Management, wildlife control	11.1	0.005	0.102 (1, 19)	0.753	−0.068 ± 0.212	0.929	0.134
Methods	3.5	0.345	10.017 (1, 19)	0.005*	0.312 ± 0.099	0.864	0.007
Morphology, physiology	2.5	—	—	—	—	—	—
Occupancy	7.8	0.476	17.269 (1, 19)	<0.001*	0.632 ± 0.152	0.911	0.054
Population dynamics	8.3	0.120	2.601 (1, 19)	0.123	−0.294 ± 0.182	0.930	0.129
Predator–prey relationships	3.3	0.004	0.079 (1, 19)	0.781	— 0.025 ± 0.089	0.959	0.515
Public health	10.0	0.041	0.822 (1, 19)	0.376	−0.158 ± 0.175	0.967	0.646
Reproduction	3.6	0.101	2.126 (1, 19)	0.161	0.167 ± 0.115	0.873	0.012
Wildlife disease	14.1	0.016	0.301 (1, 19)	0.590	0.117 ± 0.213	0.945	0.265

*Note*: Asterisks indicate significant *p* values. Data were transformed to the percentage of urban wildlife publications fitting each topic, so slopes represent the percent change per year of representation within urban vertebrate literature. The percentage of studies with focus on each subtopic over the study period, the coefficient of determination (*R* ^2^), *F*‐statistic (*F*), degrees of freedom (*df*), *p* value for regression model (*p*_1_), slope (*β*) ± the standard error (*SE*) of the slope, Shapiro–Wilk test statistic for normality (*W*), and its corresponding *p* value (*p*_2_) are reported for all regression analyses. Urban studies regarding vertebrate morphology and physiology did not appear at a noticeable frequency until 2017, so linear regression analysis was not conducted for this subtopic. Regressions for diet and methods studies were significant, though data for these regressions were not normally distributed.

**FIGURE 2:**
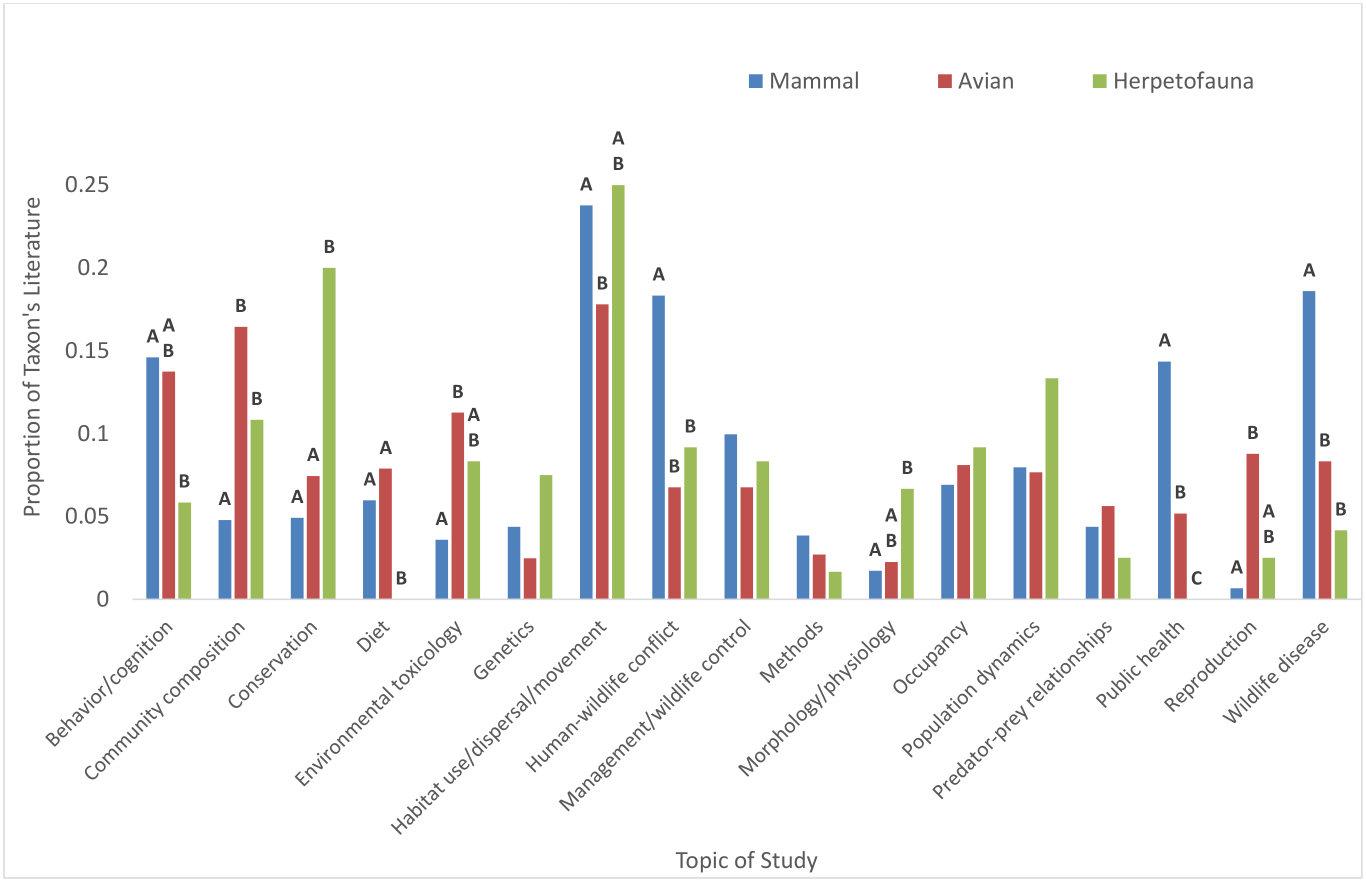
The proportion of each taxon's respective urban vertebrate literature focusing on subtopics, across the entire study period (1996–2021). Pairwise Fisher's exact tests were used to compare proportion of each taxon's urban literature for each subtopic. Significant pairwise differences within subtopics are indicated by different letter labels above bars, based on a Bonferroni‐corrected confidence level (*α* = .0167).

Our MRPP revealed that the three taxonomic groups showed separation in terms of study topics (Δ_O_ = 1.514, *T* = −30.931, *A* = 0.011, *p* < .001). All pairwise MRPPs revealed separation between all possible comparisons (herpetofauna – avian: *T* = −8.653, A = 0.005, *p* < .001; avian – mammal: *T* = −31.540, *A* = 0.009, *p* < .001; herpetofauna – mammal: *T* = −15.781, *A* = 0.006, *p* < .001). At least one significant difference existed among the percentage of studies by topic among taxa for 11 of the topics (all *p* < .016); there were no differences in proportions of studies by taxa for methods, occupancy, population dynamics, management/wildlife control, predator–prey relationships, and genetics following Bonferroni correction (all *p* ≥ .021; Figure 2, Table S1).

### 3.4 Representation of listed and non‐native species, geographies

We found no significant trend in the percent of urban wildlife studies investigating listed species (*β* = −0.113, *F* = 0.732, *df* = 1, 19, *p* = .403) nor non‐native species (*β* = 0.132, *F* = 0.338, *df* = 1, 19, *p* = .568) through the study period. Just 5.1% of publications studying urban birds looked at listed species, a significantly lower portion than mammal (10.6%; *p* = .001) and herpetofauna urban publications (13.8%; *p* = .003) studying listed species. Herpetofauna and mammal urban literature did not significantly differ in the proportion of studies investigating listed species (*p* = .327). Urban mammal studies were more likely than urban bird studies to research non‐native species (*p* < .001), at 14.8% and 6.9% of their respective groups. Urban herpetofauna research included non‐native species 8.3% of the time, which was not significantly different from either other group (herpetofauna – bird *p* = .534, herpetofauna – mammal *p* = .077).

**FIGURE 3:**
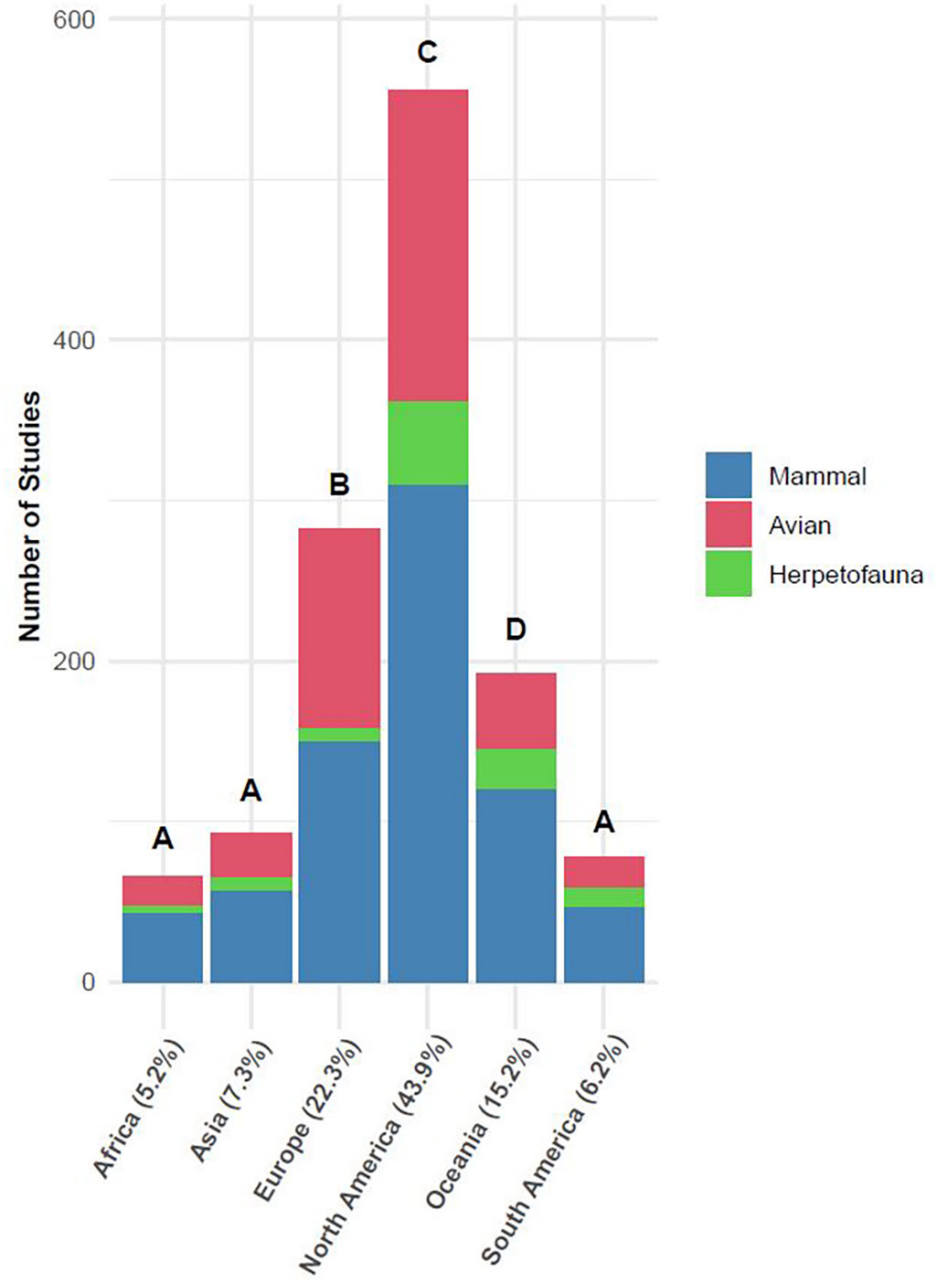
The number of urban vertebrate publications with study areas in a given continent across the study period (1996–2021). Counts that differed, based on pairwise Fisher's exact tests with a Bonferroni‐corrected confidence level (*α* = .003), are indicated by different letter labels above bars. The taxonomic representation within publications from each continent are symbolized by stacked, color bars. The values next to continent names represent the percentage of the total urban vertebrate publications that were studying areas in that continent.

**FIGURE 4:**
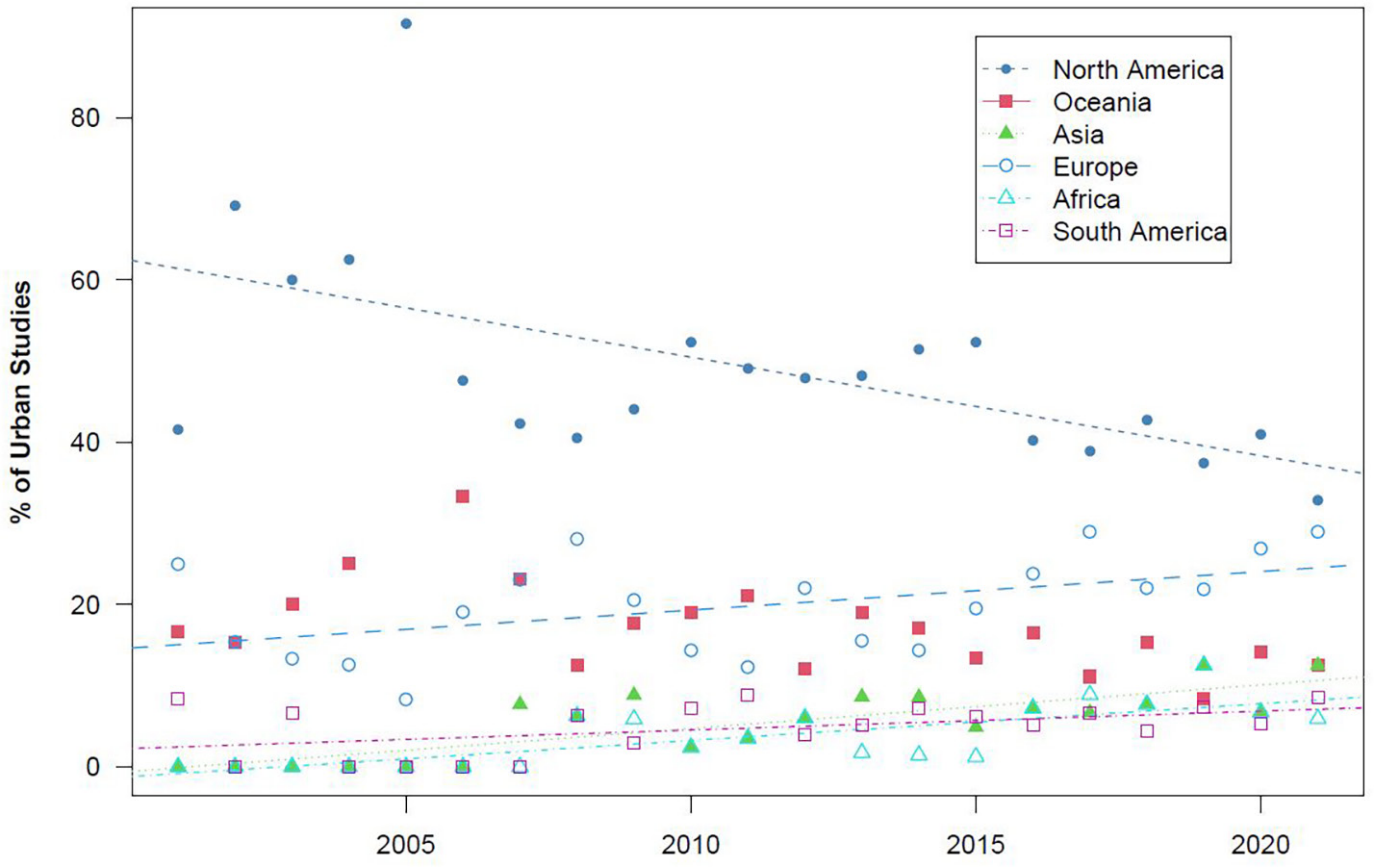
Trends in the relative percentage of urban wildlife studies by continent. Significant trends are represented by lines; only Oceania showed no significant trend (see Table S2 for full regression output).

North America had significantly more urban ecology studies than any other continent, followed by Europe and then Oceania; there were no significant differences in the number of studies among Africa, Asia, and South America (Figure 3; also see Figure 3 for taxonomic representation for literature from each continent and each continent's proportion of the urban literature). The data for North American publication rate over time was not normally distributed (*W* = 0.867, *p* = .008) due to the year 2005 being a high outlier (Figure S2). Following the removal of this data point, we found that the percentage of urban wildlife papers focused on North America decreased over time (*β* = −0.913, *F* = 11.805, *df* = 1, 18, *p* = .003). In contrast, the relative contribution of urban wildlife papers centered on Africa, Asia, Europe, and South America increased during this period (all *F* ≥ 5.334, all *p* ≤ .032), and there was no change over time for Oceania (*F* = 2.094, *df* = 1, 18, *p* = .164; Figure 4; Table S2).

## 4 DISCUSSION

We found that urban ecosystems are of growing interest in the overall terrestrial vertebrate literature, with 2.96% of publications in 2021 studying urban areas, compared to 0.71% in 2001, and urban studies are increasing within each taxon's respective body of literature. We discovered taxonomic differences in urban ecology publication rates, subtopics, and focus on listed species and non‐native species. Geographic disparities were identified that follow trends in authorship and study areas in the overall ecology and evolution literature (Hughes et al., 2023; Maas et al., 2021; Martin et al., 2012). North America was the most studied continent, while Africa, Asia, and South America were significantly less studied, however, our regression trends suggest that these taxonomic and geographic disparities are narrowing (Figures 1d and 4).

The significant breakpoint in the overall urban studies regression (Figure 1a) in 2005 was not mimicked by any individual taxon, though large, but non‐significant, increases in avian and mammalian literature at this point in time, likely combined to produce the significant increase in the rate of urban publications. This increase could have been a response to call‐to‐action publications regarding urban ecology in 1992 (Pickett et al., 1992) and 2002 (Miller & Hobbs, 2002). Pickett et al. (1992) recognized that threats to biodiversity from urbanization needed more attention in the scientific community. A decade after Pickett's insights, Miller and Hobbs (2002) determined urban studies were still lacking. Another possibility for the increased focus on urban ecology around 2005 could be that the number of humans living in urban areas surpassed that of rural areas in 2007 (Ritchie & Roser, 2018). A logical response by wildlife researchers to human migration to urban areas would be to gain a better understanding of novel urban ecosystems.

Our findings for the representation of herpetofauna (8.9%), birds (35.2%), and mammals (58.9%) within the overall urban vertebrate literature, are similar to Collins et al. (2021; herpetofauna = 5.9%, birds = 34.2%, and mammals = 59.9%; after recalculating for only terrestrial vertebrates) despite our much broader survey. In contrast, Rega‐Brodsky et al. (2022) found birds to be the most studied group in community ecology research, possibly because birds lend themselves to point counts and citizen science methods that often collect multi‐species data. We failed to detect a change in taxonomic representation in the urban literature over time (Figure 1c), as did Collins et al. (2021). However, we found the urban publication rate to be currently increasing by 0.13%/year, whereas Collins et al. (2021) found the urban wildlife publication rate (including arthropods and fish studies) currently increasing by 0.02%/year in the 16 “high impact” journals they searched while updating Magle et al. (2012). While our methods vary, we believe our estimated growth rate is more representative of the overall trend in terrestrial vertebrate sciences, as we captured a wider breadth of journals, including some explicitly focused on urban areas (e.g. Urban Ecosystems, Urban Forestry & Urban Greening), most of which were not evaluated by the regression analysis of Collins et al. (2021).

Herpetofauna are generally studied less than mammals and birds overall (Figure 1d), which also holds true when looking at taxonomic representations within the urban vertebrate literature (Figure 1c), and urban studies are less common within herpetological research than mammalian or avian literature (Figure 1b). Considering that herpetofauna are far more species rich (53% of vertebrate species) than either birds or mammals (29% and 17% of vertebrate species, respectively; IUCN, 2022), species richness does not account for the disparity observed, but herpetofauna's greater sensitivity to urbanization might (Callaghan et al., 2021; Hamer & McDonnell, 2008). However, the number of urban herpetofauna publications in 2021 was a high outlier, suggesting the current trend in urban herpetology should be monitored to determine if researchers are responding to previous findings regarding the dearth of urban herpetofauna studies. To track the research trends of other taxa and prepare for a growing wildlife‐urban interface, we assert herpetologists should direct more attention to urban ecosystems.

Being a relatively new subdiscipline, urban ecology publication trends can shift quickly. This is highlighted by the 2012 breakpoint in urban ornithology, marking an abrupt shift in urban ecology, when the publication rate for birds (currently 0.210%/year) surpassed that of mammals (0.178%/year), possibly accounting for the uptick in urban community ecology publications around that time reported by Rega‐Brodsky et al. (2022). Future breakpoints will likely occur. The high outlier in 2021 of the proportion of urban studies in herpetology (Figure S1) could be an example of such a shift, whereby the urban herpetology publication rate could be catching up with urban ornithology and urban mammalogy.

While it is still too early to analyze by breakpoint analysis, the COVID‐19 pandemic emerging in 2020 could have inflated the number of urban studies given the cancelation of field seasons and travel shutdowns. Field sites are more likely to be in protected and less‐disturbed landscapes (Martin et al., 2012; Miller & Hobbs, 2002), thus research conducted nearer to an individual's home or institution is likely to be in more urbanized areas. Additionally, many studies directly investigated the COVID‐related anthropause on wildlife behavior and movement (see Gordo et al., 2021; Silva‐Rodríguez et al., 2021). As the percentage of humans living in urban areas continues to grow, urbanized areas continue to expand, and human–wildlife interactions increase, we expect to see continued increases in urban literature.

Regarding our investigation into urban ecology subtopics, we saw that habitat use/dispersal/movement studies (herein grouped as one subtopic) to be the most prevalent focus for all vertebrate groups (Figure 2). This is possibly due to the increasing prevalence of modeling techniques, like species distribution modeling (Araújo et al., 2019; Booth, 2018; Srivastava et al., 2019), driven by an attempt to understand how climate and land use change will impact the distribution and movement of animals through fragmented habitats, as well as the ubiquity and inexpensiveness of remote cameras. Genetics and methods studies were among the least common, as fewer journals might publish these kinds of papers. Diet, predator–prey, and reproduction studies were also uncommon, possibly because of a lack of multi‐species and long‐term studies in urban ecology (Rega‐Brodsky et al., 2022).

Though decreasing over time, the rate of bird studies accounted for the highest proportion of overall vertebrate literature (Figure 1d), although mammal studies accounted for the greatest proportion of urban vertebrate literature (Figure 1c). As urban areas are densely populated with human beings, wildlife studies in these settings might be skewed towards human‐centric research objectives. We think this explains why mammals are the most studied taxa in urban ecosystems. Mammals are more likely, relative to birds and herpetofauna, to be the focus of three out of the four most prevalent subtopics: wildlife disease, behavioral/cognition, and public health, as well as for human–wildlife conflict which is the 7th most prevalent subtopic (Figure 2).

The disproportionately high focus on mammals across urban studies seem to fit the reality of human‐centric research needs, as most human–wildlife communicable diseases come from mammals (of vertebrate groups and not including insects; Wolfe et al., 2007). Other reviews have also shown a disproportionately high focus on mammals in human–wildlife conflict literature (Seoraj‐Pillai & Pillay, 2016; Taylor & Goldingay, 2010). This potential bias is not just among researchers, as drivers are more concerned with collisions with large mammals than herpetofauna or birds (Crawford & Andrews, 2016), even though other taxa are more likely to be involved in vehicle‐wildlife incidents (Glista et al., 2008; Kioko et al., 2015). This perception likely arises from mammals being the taxa most likely to cause fatal accidents in wildlife‐vehicle collisions in many countries (Ramp & Roger, 2008; Rowden et al., 2008; Sáenz‐de‐Santa‐María & Tellería, 2015; Vanlaar et al., 2019). Given these dynamics, focus on mammals rather than other groups might be expected where human lives and property are concentrated.

However, while mammals are the most likely group to cause fatal vehicle accidents, snakes kill more people (100,000/year according to Gutiérrez et al., 2017) across the globe than any other animal group besides mosquitoes, who kill indirectly by being disease vectors (Chandrasegaran et al., 2020), and other humans (UN Office on Drugs and Crime, 2019). While rural dwellers bitten by snakes can suffer more consequences due to their remoteness to medical facilities (Hong et al., 2020), snakebites can be common in urban locations (Hauptfleisch et al., 2021; Yue et al., 2019). Despite this, we found mammals were twice as likely to be the focus of human–wildlife conflict research in urban areas than herpetofauna, and we found no urban public health studies relating to herpetofauna. This apparent knowledge gap could be caused by disproportionately low publication rates of urban studies in Asia, Africa, and South America (Figure 3), where most lethal snakebites occur (Gutiérrez et al., 2017). We argue more focus should be directed towards dynamics between urbanization and herpetofauna related conflicts and public health concerns, especially in areas where snakebites are most lethal.

Listed species might be less common than least‐concern species near urban areas due to increased sensitivity to urbanization (e.g., Pfeifer et al., 2017), though urbanization is known to be one of the main drivers of extinction risk for vertebrates (Keinath et al., 2017; McDonald et al., 2008), making conservation studies where listed species interface with urban areas crucial. For each vertebrate group, the percentage of urban studies investigating listed species was lower than the percentage of species that are listed (a disparity of about 16% for mammals, 8% for birds, and 11% for herpetofauna; IUCN, 2022). More conservation studies in urban areas, as well as additional efforts by ecologists to tie biodiversity to ecosystem services (Rega‐Brodsky et al., 2022), could help reduce the negative impact of urbanization on rare species.

Although herpetofauna were generally understudied in urban areas, this group was most likely to be the focus of urban conservation studies, proportionally speaking (Figure 2). Urban herpetology papers were significantly more likely than urban ornithology papers to be studying listed species. A disproportionately high extinction risk for the class amphibia exists; of IUCN‐assessed species, 41% of amphibians, 27% of mammals, 21% of reptiles, and 13% of birds are threatened by extinction (IUCN, 2022), likely explaining the high frequency of herpetology studies investigating listed species. Herpetofauna are particularly sensitive to land conversion and fragmentation (Keinath et al., 2017; Powers & Jetz, 2019), and urbanization is thought to be the most detrimental of land use changes for herpetofauna species richness (Cordier et al., 2021). Therefore, we find it intuitive that urban herpetology was most likely to focus on habitat use, dispersal, and movement studies (aggregated into a single group in this review; Figure 2), as these topics are coupled with the conservation concerns of this group.

Urban mammalogy was more likely than urban ornithology to study non‐native species, while urban herpetology did not differ from either group, possibly related to urban mammal studies more often investigating wildlife disease and public health topics. There could be a particularly heightened awareness surrounding non‐native mammal invasions, as mammals are more associated with human–wildlife communicable diseases (Wolfe et al., 2007). Domestic cats (*Felis catus*) and rats (most commonly *Rattus rattus* and *R. norvegicus*) were the most studied non‐native species, accounting for 34.7% and 16.3%, respectively, of the urban studies researching non‐native species. While domesticated animals might not be considered wildlife, we included these studies because feral populations of such animals often interact with their surrounding ecosystems in a detrimental fashion, such as disease transmission and predation of native species (Baker et al., 2018; Davis et al., 2018; Legge et al., 2017). Since these invasions appear to be a geographically ubiquitous issue (Dueñas et al., 2021; Medina et al., 2011), we assert that the threat to biodiversity posed by these common non‐native species be prioritized, globally, by urban planners and stakeholders.

Regarding nativeness, researchers should explicitly state the biogeographical context of the populations they are investigating, as this context should not be assumed common knowledge for global audiences. This highlights a possible need for consensus regarding explicit definitions of different population histories and sources (e.g., non‐native versus naturalized species), a possibly convoluted realm (see Blackburn et al., 2011; Crees & Turvey, 2015) of wildlife sciences that will only be exacerbated by climate change induced range shifts predicted for many species (Parmesan & Yohe, 2003; Tingley et al., 2009; Widmer et al., 2022).

We found 67% of urban vertebrate publications studied places in North America or Europe, while only 18% came from South America, Asia, or Africa (Figure 3). Similar geographic disparities have been reported for publications in urban ecology (Collins et al., 2021; Rega‐Brodsky et al., 2022) and ecology overall (Di Marco et al., 2017; Hughes et al., 2023; Maas et al., 2021; Martin et al., 2012). Despite being the least studied, South America, Asia and Africa are the most biodiverse areas on earth (Olson et al., 2001), and the latter two continents are projected to experience the greatest amount of urbanization in the coming decades (Awumbila, 2017), highlighting a need to increase urban studies in those areas. However, these regions are exhibiting slight increases in their representation within urban vertebrate ecology (Figure 4). While North American studies are most common, their relative contribution to the urban literature is trending downward at a rate of 0.913%/year (Figure 4). We acknowledge the limitation that we searched only for publications that were published in English, which may have inflated studies from North America where English is the first language of many researchers.

The geographic disparities we found could improve with some course correction within the field. Additional barriers to entry exist for wildlife researchers based on their location (Nagendra et al., 2018), and most urban ecologists currently study cities local to them (Rega‐Brodsky et al., 2022). Increasing collaboration between researchers in underrepresented and overrepresented regions could better allocate resources geographically and could aid in decolonizing the field (see Trisos et al., 2021). Diversity, equity and inclusion efforts among research institutions and funding sources could promote a more geographically even distribution of urban wildlife research in the future by supporting more work in currently underrepresented places. We encourage future reviews to monitor the progress of urban ecology, and suggest future inquiries attempt to broaden their searches by including non‐English language publications and searching multiple databases. In the interim, increased focus on threatened species, the dynamics between urbanization and non‐native invasions, and herpetofauna will make the growing field of urban ecology more robust and positioned to benefit natural ecosystems and human society alike.

Our findings recognize that wildlife research has shown an increasing focus on urban ecosystems over the last few decades. With the proportion of human beings living in urban areas (UN Department of Economic and Social Affairs, 2018) and the proportion of developed land (Chen et al., 2020) projected to continue growing, globally, this increased focus on urban ecosystems tracks a natural demand in ecological and conservation research. While wildlife biology is meeting the moment in one respect, there are some current disparities regarding the geographic distribution of this research and the taxa being studied, as well as the prevalence of studying important categories of wildlife (e.g., non‐native and listed species).

## AUTHOR CONTRIBUTIONS

**Travis A. Rainey:** Conceptualization (supporting); data curation (lead); formal analysis (lead); investigation (equal); methodology (equal); visualization (lead); writing – original draft (supporting); writing – review and editing (equal). **Alaini C. Schneider:** Conceptualization (lead); data curation (supporting); formal analysis (supporting); investigation (equal); methodology (equal); writing – original draft (lead); writing – review and editing (equal). **Carson J. Pakula:** Data curation (supporting); formal analysis (supporting); investigation (equal); methodology (equal); writing – review and editing (equal). **Bradley J. Swanson:** Conceptualization (supporting); formal analysis (supporting); investigation (equal); methodology (equal); project administration (lead); writing – original draft (supporting); writing – review and editing (supporting).

## CONFLICT OF INTEREST STATEMENT

The authors declare that there are no conflicts of interest.

## Supporting information


Figure S1:

**Data S1:** Supporting information

## Data Availability

Data and code used in this study are provided in the Supporting Materials.
