# Supplementary material for: As human societies urbanize, so does ecology; taxonomic, geographic, and other research trends in urban vertebrate ecology
Source: Ecol Evol. 2024 May 20;14(5):e11439. doi: 10.1002/ece3.11439 (PMC11106554; doi:10.1002/ece3.11439)
Supplement: Supplementary file 1 — Figure S1: [file ECE3-14-e11439-s001.docx]

As human societies urbanize, so does ecology; taxonomic, geographic, and other research trends in urban vertebrate ecology — Supplementary Tables and Figures

Table S1: Fisher’s exact test p values for pairwise comparisons between the proportion of urban publications covering subtopics between taxonomic groups (e.g., the proportion of urban avian publications dealing with occupancy compared to the proportion of urban mammal studies dealing with occupancy). Asterisks indicate significant differences based on a Bonferroni-corrected significance level (α = 0.0167). Codes are given for taxa (HE for herpetofauna, AV for birds, MA for mammals).

|  | P values (*p*) | | |
| --- | --- | --- | --- |
| Topic | HE - AV | MA - AV | HE - MA |
| Behavior/cognition | 0.017 | 0.733 | 0.009* |
| Community composition | 0.153 | < 0.001* | 0.016* |
| Conservation | < 0.001* | 0.076 | < 0.001* |
| Diet | < 0.001 | 0.231 | 0.002* |
| Environmental toxicology | 0.408 | < 0.001* | 0.026 |
| Genetics | 0.021 | 0.111 | 0.164 |
| Habitat use/dispersal/movement | 0.090 | 0.016* | 0.818 |
| Human-wildlife conflict | 0.427 | < 0.001* | 0.013* |
| Management/wildlife control | 0.550 | 0.072 | 0.740 |
| Methods | 0.744 | 0.327 | 0.296 |
| Morphology/physiology | 0.034 | 0.521 | 0.004* |
| Occupancy | 0.711 | 0.492 | 0.347 |
| Population dynamics | 0.069 | 0.912 | 0.079 |
| Predator-prey relationships | 0.235 | 0.333 | 0.461 |
| Public health | 0.007* | < 0.001* | < 0.001* |
| Reproduction | 0.018 | < 0.001* | 0.084 |
| Wildlife disease | 0.169 | < 0.001* | < 0.001* |

Table S2: Regression results for the percentage of urban wildlife publications studying areas in each continent. Asterisks indicate significant p values. Coefficient of determination (*R^2^*), F-statistic (*F*), degrees of freedom (df), p value for regression model (*p_1_*), slope (*β*) ± the standard error (SE) of the slope, Shapiro-Wilk test statistic for normality (*W*), and its corresponding p value (*p_2_*) are reported for all regression analyses. The data for North America had non-normally distributed data due to a high outlier in 2005 (see text for details), so this year was removed for that regression analysis.

| Continent | *R^2^* | *F* (df) | *p_1_* (*F*) | *β* ± SE | *W* | *p_2_* (Shapiro Wilk) |
| --- | --- | --- | --- | --- | --- | --- |
| Africa | 0.572 | 25.370 (1, 19) | < 0.001* | 0.453 ± 0.090 | 0.987 | 0.990 |
| Asia | 0.658 | 36.620 (1, 19) | < 0.001* | 0.538 ± 0.089 | 0.922 | 0.094 |
| Europe | 0.236 | 5.883 (1, 19) | 0.025* | 0.475 ± 0.196 | 0.970 | 0.738 |
| North America | 0.396 | 11.805 (1,18) | 0.003* | -0.913 ± 0.266 | 0.986 | 0.987 |
| Oceania | 0.099 | 2.094 (1, 19) | 0.164 | -0.337 ± 0.233 | 0.903 | 0.040 |
| South America | 0.219 | 5.334 (1, 19) | 0.032* | 0.233 ± 0.101 | 0.963 | 0.582 |





Figure S1: Residual plot for the simple linear regression model of the publication rate of urban herpetofauna studies across the study period (2001 – 2021; there were no urban herpetology papers detected prior to 2001), showing 2001 and 2021 as high outliers (i.e., datapoints with a corresponding residual 2 standard deviations from the mean).





Figure S2: Residual plot for the simple linear regression model of the proportion of urban studies with North American study areas across the study period (2001 – 2021; there was an insufficient number of urban studies per year, prior to 2001, for relative proportion calculations), showing 2005 as a high outlier (i.e., datapoint with a corresponding residual 2 standard deviations from the mean).
